# Research on Airplane and Ship Detection of Aerial Remote Sensing Images Based on Convolutional Neural Network

**DOI:** 10.3390/s20174696

**Published:** 2020-08-20

**Authors:** Changqing Cao, Jin Wu, Xiaodong Zeng, Zhejun Feng, Ting Wang, Xu Yan, Zengyan Wu, Qifan Wu, Ziqiang Huang

**Affiliations:** School of Physics and Optoelectronic Engineering, Xidian University, 2 South Taibai Road, Xi’an 710071, China; chqcao@mail.xidian.edu.cn (C.C.); xdzeng@xidian.edu.cn (X.Z.); zhjfeng@mail.xidian.edu.cn (Z.F.); tingw563355@gmail.com (T.W.); yanxu_xy@163.com (X.Y.); zengyanw0@163.com (Z.W.); jiji15900@163.com (Q.W.); zqhuang97@163.com (Z.H.)

**Keywords:** target detection, aerial remote sensing image, DOTA dataset, YOLOv3

## Abstract

The wide range, complex background, and small target size of aerial remote sensing images results in the low detection accuracy of remote sensing target detection algorithms. Traditional detection algorithms have low accuracy and slow speed, making it difficult to achieve the precise positioning of small targets. This paper proposes an improved algorithm based on You Only Look Once (YOLO)-v3 for target detection of remote sensing images. Due to the difficulty in obtaining the datasets, research on small targets for complex images, such as airplanes and ships, is the focus of research. To make up for the problem of insufficient data, we screen specific types of training samples from the DOTA (Dataset of Object Detection in Aerial Images) dataset and select small targets in two different complex backgrounds of airplanes and ships to jointly evaluate the optimization degree of the improved network. We compare the improved algorithm with other state-of-the-art target detection algorithms. The results show that the performance indexes of both datasets are ameliorated by 1–3%, effectively verifying the superiority of the improved algorithm.

## 1. Introduction

With the advent of the information age, remote sensing technology has remedied the problem of the limited coverage of traditional ground detection and serious lack of related detection data through rapid air-to-ground information acquisition and target detection. Due to its significant advantages of flexible maneuverability, high resolution, and optional observation range, aerial remote sensing provides a new method for target detection. Airplanes and ships are vital strategic resources and means of transportation in both military and civilian fields. Therefore, they are of great significance to the research of remote sensing image detection. Compared with road vehicle detection, remote sensing images of airplanes and ships have complex backgrounds with diverse targets and small sizes, so target detection is more challenging in this context.

Before the rise of convolutional neural networks, aerial remote sensing images relied on traditional algorithms for target detection. Traditional target detection algorithms mainly include edge detection algorithms [1], such as the Roberts algorithm [2]; threshold segmentation methods [3], such as the Otsu threshold segmentation algorithm [4]; visual saliency detection algorithms, such as the ITTI algorithm [5]. The first two algorithms complete the detection task by detecting the strong contrast and the difference in gray value between the target and the image background, respectively, and the latter algorithm obtains the positioning through the difference in imaging angle. However, for complex remote sensing images, too many interference elements lead to poor positioning accuracy.

Based on the above problems, convolutional neural networks (CNNs) have replaced traditional target detection algorithms to meet the small target detection of aerial remote sensing images with complex backgrounds. CNN-based target detection algorithms can be divided into two categories: The first being region-based target detection algorithms, forming the two-stage algorithms represented by R-CNN [6,7,8]. The detection accuracy of this algorithm is high, but the speed is slow. The second type is the regression-based target detection algorithm, forming the one-stage algorithms represented by You Only Look Once (YOLO) [9,10,11] and SSD (Single Shot multibox Detector) [12]. This algorithm converts the detection problem into a regression problem and the speed is significantly accelerated. The CNN network has outstanding advantages in remote sensing images and can successfully complete target positioning and classification tasks. Deng et al. [13] presented a method based on an enhanced deep CNN, which followed the general process of “CNN feature extraction + region suggestion + region classification” and successfully implemented a test of large-scale Google Earth images. Long et al. [14] developed an object localization framework based on CNN in remote sensing images. Yu et al. [15] introduced a bilinear convolutional neural network model for scene classification, which greatly improved the performance and accuracy of remote sensing image classification tasks. Focusing on the problem of target detection in remote sensing images, Yao et al. [16] proposed an integrated model based on Faster R-CNN to detect chimneys and condensation towers in high-resolution remote sensing images. Tang et al. [17] used a Faster R-CNN-based network to monitor vehicle targets in remote sensing images in real time. Facing smaller targets and more difficult airplane and ship missions, Zhang et al. [18] proposed a weakly supervised learning framework based on coupled convolutional neural networks for airplane detection. Xu et al. [19] proposed a remote sensing image airplane detection method that used multilayer feature fusion in fully convolutional neural networks. Zou et al. [20] designed the Singular Value Decomposition network (SVDNet) for ship detection based on the convolutional neural network and the SVD algorithm. Wang et al. [21] studied a convolutional neural network-based renormalization method to realize ship detection with very high resolution (VHR) remote sensing images. Zhang et al. [22] designed a Deconv R-CNN model through a network with a deconvolution layer after the last convolution layer of the basic network for airplane and ship detection. The above methods are more suitable for large-scale targets with high contrast in natural scenes, but in the case of complex backgrounds and the detection of small targets, the detection results are less accurate. 

The detection accuracy and real-time performance of the YOLO series of algorithms have significant advantages over other algorithms, among which the YOLOv3 algorithm is considered the best. This paper aims to improve the network based on YOLOv3 to further meet the detection requirements for small targets. Training based on a convolutional neural network requires a large number of samples. In order to make up for insufficient data, we screened specific types of training samples from the DOTA (Dataset of Object Detection in Aerial Images) dataset and trained the detection network of aerial remote sensing images through the synthetic dataset. The detection accuracy of small targets in the network model was relatively high. We chose small targets in two different complex backgrounds (i.e., airplanes and ships) to boost the optimization of the network model and improved the accuracy requirements of the network model. To solve the problem of low detection accuracy of small targets, a detection scale was added to the deep features of the network to obtain a smaller receptive field to enhance the sensitivity to small targets. During the training process, the imbalance of positive and negative samples may lead to data overfitting. L2 regularization was appended to the network to improve the overall loss function and enhance the anti-interference ability of the network model. The experimental results show that the improved network has higher accuracy than the previous detection algorithms.

## 2. Related Work

### 2.1. YOLOv3 Network Analysis

The YOLO series algorithm is a typical end-to-end network, which can directly obtain the target’s bounding box information and class probability through a forward operation. Compared with the R-CNN series of the two-stage algorithms, the candidate region mechanism and the detection layer are integrated in the convolutional network, which can greatly improve the detection speed.

#### 2.1.1. Network Structure

The target detection algorithm based on YOLO defines the detection problem as a single regression problem, that is, a single neural network can realize the prediction of multiple bounding boxes and class probabilities on the entire image at the same time through a forward operation. Based on the previous detection algorithm, YOLOv3 was proposed in 2018 with the introduction of multi-scale target detection through the feature pyramid network (FPN) [23], and proposed a new feature extraction network, Darknet-53, after borrowing the structure of the residual network (ResNet) [24].

**Figure 1:**
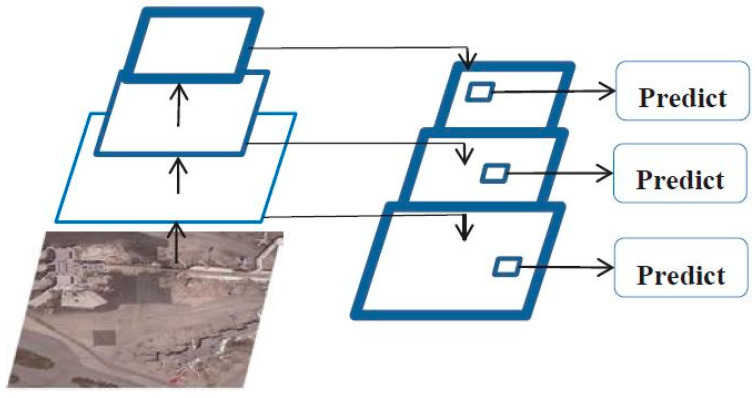
The schematic diagram of multi-scale prediction.

The Darknet-53 network includes 53 convolutional layers and five residual modules. Each residual module is composed of 1 × 1 and 3 × 3 convolutional layers and a shortcut connection. The convolutional structure consists of convolutional layers, batch normalization, and nonlinear activation function—Leaky Relu. Compared with the Darknet-19 network, the pooling layer is replaced by a 3 × 3 convolutional layer with a step size of 2, which retains more feature information during the down-sampling process. The residual module references the network connection mode of the residual skip layer in ResNet to solve the gradient disappearance or explosion caused by the deep network structure and promotes YOLOv3 to use deeper feature extraction layers to extract feature information with a wider range and higher accuracy for targets detection. At the same time, YOLOv3 introduces multi-scale prediction through the FPN to enhance the sensitivity of small targets. The structure diagram of multi-scale prediction is shown in Figure 1.

**Figure 2:**
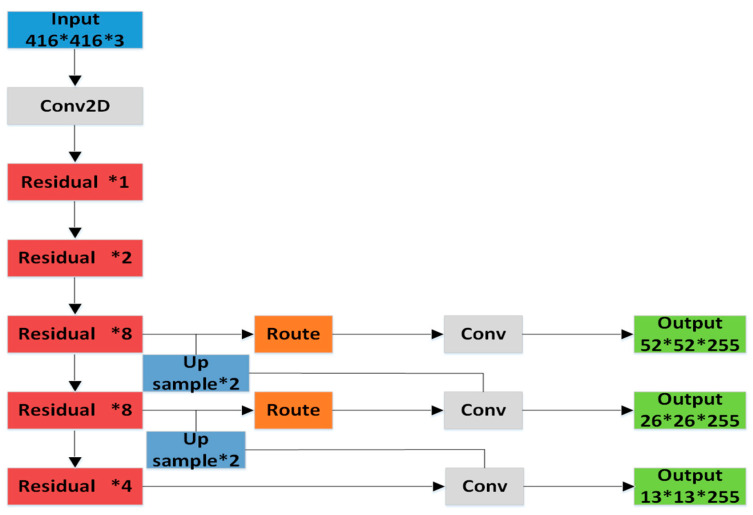
The network of You Only Look Once (YOLO)-v3.

Figure 1 shows that the FPN uses feature maps of different sizes for fusion to achieve target detection. With the deepening of the network, the size of the output feature map of the convolution at each layer is gradually reduced, and the target detection work is completed after the fusion of feature maps in different sizes. The network up-samples each layer of the feature pyramid from top to bottom, and then connects it with the feature map of the next layer horizontally to obtain the new enhanced feature map. The new feature map contains not only the rich semantic information output by the deep network, but also the accurate target position information output by the shallow network. Separate target detection is performed on these newly acquired feature maps. In YOLOv3, feature maps with three different sizes of 13 × 13, 26 × 26, and 52 × 52 are applied to construct feature pyramids, which are used to detect large, medium, and small-sized targets, respectively. Multi-scale prediction makes YOLOv3 more sensitive to weak targets and significantly boosts its detection ability. The input images undergo a series of operations to finally obtain three output feature maps of different sizes, namely, 13 × 13 × 255, 26 × 26 × 255, and 52 × 52 × 255. Each grid cell contains three anchor boxes. Therefore, the output tensor 255 is 3 × (4 + 1 + 80), which corresponds to four bounding box coordinates, one confidence prediction and 80 class predictions of the object. The structure diagram of YOLOv3 is shown in Figure 2.

#### 2.1.2. Testing Process

**Figure 3:**
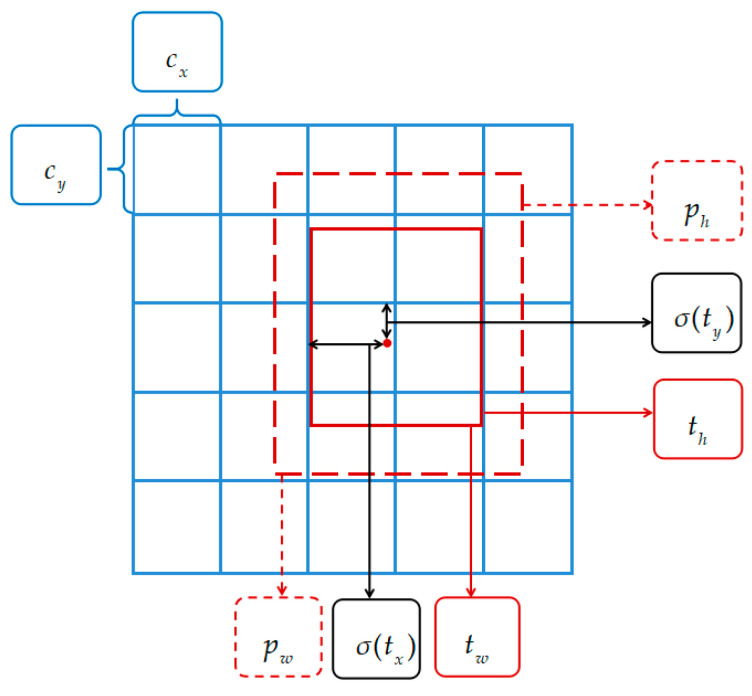
The final prediction.

The YOLOv3 network resizes the input image to a size of 416 × 416 before feature extraction, and then divides it into S × S grids for prediction. The center of the target will fall within the grid responsible for detecting the target. Each grid can predict B bounding boxes, and each bounding box contains information for five parameters. The network generates S × S × B bounding boxes on the feature map and directly performs a regression on the generated bounding boxes. The modified predicted bounding box coordinates of the YOLOv3 network can be calculated by Equation (1) and the schematic diagram is shown in Figure 3.

(1) $$\begin{array}{l} b_{x}=\sigma (t_{x})+c_{x}\\ b_{y}=\sigma (t_{y})+c_{y}\\ b_{w}=p_{w}e^{t_{w}}\\ b_{h}=p_{h}e^{t_{h}} \end{array}\},$$

where $b_{x}$, $b_{y}$ is the coordinate of the center point of the modified bounding box, respectively; $b_{w}$, $b_{h}$ is width and height of the modified bounding box, respectively; $t_{x}$, $t_{y}$ is the offset of the target center point relative to the upper left corner of the grid where the point is located; $t_{w}$, $t_{h}$ is the width and height of the predicted bounding box, respectively; $c_{x}$, $c_{y}$ is the offset of the gird relative to the upper left; $p_{w}$, $p_{h}$ is the width and height of the anchor box, respectively. The sigmoid function is used to set the value range between (0, 1) and control the offset of the target center to be within the corresponding grid unit to ensure that there is no over-offset.

To prevent the redundancy of the predicted bounding box, it is necessary to perform confidence calculation and set the confidence threshold on each predicted bounding box. When the confidence score is above the threshold, it is reserved for regression and otherwise discarded. The confidence is composed of two parts: one is the probability of the existence of the target in the grid, expressed by $P_{r}(\mathrm{object})$; when there is a target to be detected in the grid, $P_{r}(\mathrm{object})=1$, otherwise it is 0. The other part refers to the accuracy of the predicted bounding box, which is defined as follows:

(2) $$C_{conf}=P_{r}(\mathrm{clas}s_{i}|\mathrm{object})\times P_{r}(\mathrm{object})\times {\mathrm{IOU}}_{pred}^{truth}=P_{r}(\mathrm{class})\times {\mathrm{IOU}}_{pred}^{truth},$$

where $C_{conf}$ refers to the class-specific confidence scores for each box; $P_{r}(\mathrm{clas}s_{i}|\mathrm{object})$ refers to the probabilities of predicting *C* conditional class in each grid cell (*i* = 1, 2,..., *C*); $P_{r}(\mathrm{object})\times {\mathrm{IOU}}_{pred}^{truth}$ refers to the confidence score; and Intersection over Union (${\mathrm{IOU}}_{pred}^{truth}$) refers to the intersection ratio of the bounding box area and the ground truth box area.

The non-maximum suppression (NMS) algorithm can determine the degree of coincidence of the remaining boxes, and the predicted box with higher confidence is retained as the target detection box. The predicted bounding box is composed of three loss functions:

(3) $$loss{\left(object\right)}_{YOLOv3}=loss_{1}-loss_{2}-loss_{3},$$

(4) $$\begin{array}{l} loss_{1}=\lambda_{coord}\sum_{i=0}^{S\times S}\sum_{j=0}^{B}I_{ij}^{obj}[{\left(x_{i}-{\hat{x}}_{i}\right)}^{2}+{\left(y_{i}-{\hat{y}}_{i}\right)}^{2}]+\lambda_{coord}\sum_{i=0}^{S\times S}\sum_{j=0}^{B}I_{ij}^{obj}(2-w_{i}\times h_{i})[{\left(w_{i}-{\hat{w}}_{i}\right)}^{2}+{\left(h_{i}-{\hat{h}}_{i}\right)}^{2}]\\ loss_{2}=\lambda_{obj}\sum_{i=0}^{S\times S}\sum_{j=0}^{B}I_{ij}^{obj}[{\hat{C}}_{i}\log(C_{i})+(1-{\hat{C}}_{i})\log(1-C_{i})]+\lambda_{noobj}\sum_{i=0}^{S\times S}\sum_{j=0}^{B}I_{ij}^{noobj}[{\hat{C}}_{i}\log(C_{i})+(1-{\hat{C}}_{i})\log(1-C_{i})]\\ loss_{3}=\sum_{i=0}^{S\times S}\sum_{j=0}^{B}I_{ij}^{obj}\sum_{c\in classes}\left[{\hat{p}}_{i}(c)\log(p_{i}(c))+(1-{\hat{p}}_{i}(c))\log(1-p_{i}(c))\right] \end{array}\},$$

where $loss_{1}$ is the loss of the predicted bounding box; $loss_{2}$ is the loss of predicted confidence; $loss_{3}$ is the loss of the predicted class; ∧ indicates the true value, otherwise it is the predicted value; $x_{i}$, $y_{i}$, $w_{i}$, and $h_{i}$ refer to the four coordinate values of the *i*-th bounding box; λ refers to the weight of the loss; $C_{i}$ refers to the confidence of the *i*-th bounding box; $p_{i}(c)$ refers to the class probability of the *i*-th bounding box.

### 2.2. Improvement of YOLOv3

#### 2.2.1. Detection Scale

**Figure 4:**
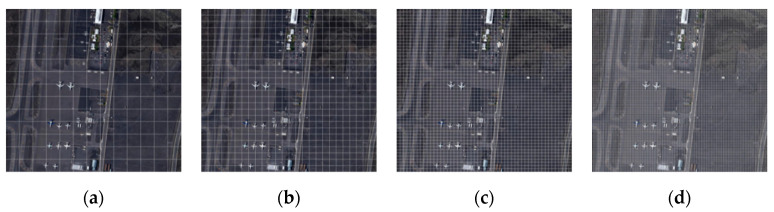
Receptive field of the same aerial remote sensing image at different scales: (**a**) 13 × 13; (**b**) 26 × 26; (**c**) 52 × 52; (**d**) 104 × 104.

The YOLOv3 network introduces a feature pyramid structure to perform multi-scale prediction on the target, effectively combining semantic information from the deep network with feature information from the shallow network to enhance the target detection accuracy. However, it still experiences difficulties in detecting medium and small targets. Here, the MyPlane and MyShip datasets used for target detection are aerial remote sensing image datasets. The targets to be detected, namely, airplanes and ships, are weak targets based on the entire picture. Therefore, we subjoined a 104 × 104 detection scale to the improved model to enhance the sensitivity and detection ability of the network to small targets. This operation can make the receptive field of the network model smaller, which is conducive to the detection of small airplane and ship targets in the background of aerial remote sensing. However, the height of aerial remote sensing imagery is not fixed, and the existing targets have relatively large targets to be detected. Therefore, the improved model in this paper continues to retain the detection work at the scale of 13 × 13. The receptive fields at the scale of 13 × 13, 26 × 26, 52 × 52, and 104 × 104 on the same aerial remote sensing image are shown in Figure 4.

**Figure 5:**
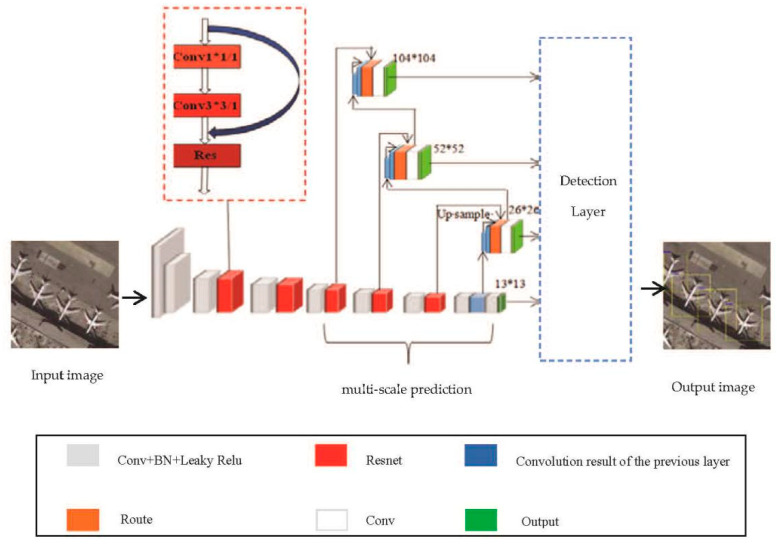
The structure of the improved network.

The improved network model increased the output of 104 × 104 × 255 with a smaller receptive field to enhance the detection ability using small targets. The improved network model graph is shown in Figure 5.

#### 2.2.2. Loss Function

In general, the mathematical model parameters obtained by the network fitting are kept to the minimum. When the new sample data processed by the network destroys the previous sample distribution with the increase of sample data, the existing mathematical model needs to be corrected. However, due to the relatively small number of parameters, the extent of modification of the entire network is actually not large and the whole network has a strong anti-disturbance capability. L2 regularization is used to avoid the occurrence of overfitting by obtaining smaller parameter weights. The expression of L2 regularization is as follows:

(5) $$J=J_{0}+\lambda \sum_{\omega }\omega^{2},$$

where $J_{0}$ represents the initial loss function, λ is the regularization parameter, and ω is the weight vector. L2 regularization is equivalent to adding a penalty term to the initial loss function. Assuming that the parameter to be sought is θ, the loss function in linear regression is as follows:

(6) $$J(\theta )=\frac{1}{2m}{\sum_{i=1}^{m}\left(h_{\theta }(x^{\left(i\right)})-y^{\left(i\right)}\right)}^{2},$$

(7) $$h_{\theta }(x)=\theta_{0}x_{0}+\theta_{1}x_{1}+L+\theta_{n}x_{n},$$

The gradient descent method is adopted to reduce the entire loss function, that is, to adjust the parameter θ in the opposite direction of the gradient, as follows:

(8) $$\frac{\partial }{\partial \theta_{j}}J(\theta )=\frac{1}{m}\sum_{i=1}^{m}\left(h_{\theta }(x^{\left(i\right)})-y^{\left(i\right)}\right){x_{j}}^{\left(i\right)},$$

(9) $$\theta_{j}=\theta_{j}-a\frac{1}{m}\sum_{i=1}^{m}\left(h_{\theta }(x^{\left(i\right)})-y^{\left(i\right)}\right){x_{j}}^{\left(i\right)},$$

(10) $$\theta_{j}=\theta_{j}\left(1-a\frac{\lambda }{m}\right)-a\frac{1}{m}\sum_{i=1}^{m}\left(h_{\theta }(x^{\left(i\right)})-y^{\left(i\right)}\right){x_{j}}^{\left(i\right)},$$

where Equation (9) is the iterative equation of the initial loss function, and Equation (10) is the iterative equation after adding L2 regularization. By comparing the two equations, θ, after increasing L2 regularization, will first be multiplied by (1−aλ/m) during each iteration. Since this factor is less than 1, the parameter $\theta_{j}$ continuously decreases. Based on the above theory, adding the L2 regularization term to the loss function of YOLOv3 can enhance the interference ability of the network and avoid overfitting.

(11) $$loss_{L2}=loss+\frac{\lambda }{2N}\sum_{\omega }\omega^{2},$$

where $loss_{L2}$ refers to the improved loss function, loss refers to the loss function of the original YOLOv3 network, and $\left(\lambda /2N\right)\sum_{\omega }\omega^{2}$ represents the L2 regularization term, where N is a constant.

## 3. Experiments

### 3.1. Network Training

The background of aerial remote sensing images is complex, which makes data acquisition extremely difficult. Training based on the convolutional neural network requires a large number of samples, but the data of aerial remote sensing images is insufficient. It is difficult to directly detect images using existing datasets. 

#### 3.1.1. Dataset Creation

Due to the specificity of aerial remote sensing images, the detection accuracy of small targets in the network model is relatively high. We chose small targets in two different complex backgrounds (airplane and ship) to assess the optimization degree of the network model and improve the accuracy requirements of the network model. Currently, the publicly known datasets of aerial remote sensing images are as follows: the NWPU VHR-10 dataset produced by Northwestern Polytechnical University, and the RSOD dataset and DOTA dataset produced by Wuhan University. Among them, the DOTA dataset has the best sample image quality at present, and the image size is generally around 4000–5000 pixels. At the same time, the DOTA dataset has 15 types of targets, including airplanes and ships, totaling more than 180,000 specific targets. This paper used the DOTA dataset to make MyPlane and MyShip datasets. Since the production process of the two sets is similar, we took the MyPlane dataset as an example. The main process was as follows:

- We used the code to automatically traverse all sample images in the DOTA dataset (containing two or more airplane targets) and filtered out the .txt annotation files.
- The excessively large size of sample images from the DOTA dataset leads to excessive compression and a large amount of information will be lost when they are sent directly to the network. Therefore, each sample image obtained was cropped into several 1024 × 1024 size images, and the corresponding .txt annotation file was updated. If the intersection ratio between the divided target and the original target was greater than 0.7, the current target was retained; otherwise it was discarded.
- We repeated the above steps to reduce the dataset until the required sample set was obtained and the preliminary preprocessing operation was completed.

**Figure 6:**
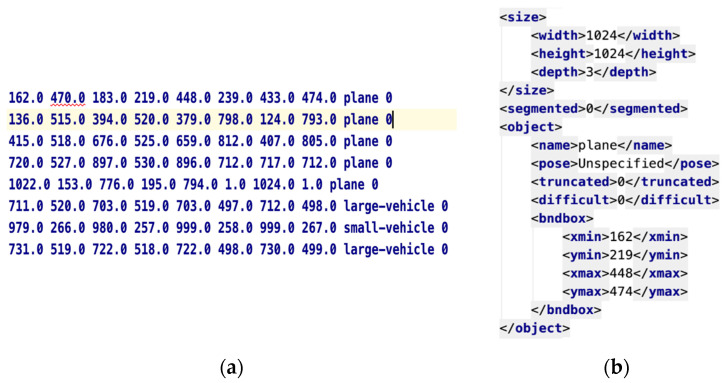
Annotation file. (**a**) DOTA (Dataset of Object Detection in Aerial Images) dataset; (**b**) the dataset used in this paper.

The annotation file type of images in DOTA dataset is the .txt file type, where the data annotation format is OBB, which is determined by the coordinate position: $\left(x_{1},y_{1}\right)$, $\left(x_{2},y_{2}\right)$, $\left(x_{3},y_{3}\right)$, $\left(x_{4},y_{4}\right)$. It is visualized as an ordinary quadrilateral in Figure 6. The final annotation file of the MyPlane datasets in this paper is the common VOC type, that is, the annotation file is in .xml format. The target position coordinates are in HBB format, which are determined by the value: $x_{\max}$, $y_{\max}$, $x_{\min}$, $y_{\min}$. It is visualized as a rectangular box in Figure 6. We used the code to convert the .txt file in OBB format into the .xml file in HBB format and deleted the annotation information of other non-airplane targets. Finally, the dataset was amplified by means of rotation angle and mirror flipping to increase the number of training samples and avoid overfitting.

Figure 6 clearly shows the annotation file of DOTA dataset and the dataset used in this paper. The production process of the entire dataset was based on the step-by-step implementation of Python code, which fully utilized the accuracy and effectiveness of the information marked by the DOTA dataset.

#### 3.1.2. Reset Anchor Box Parameters

**Figure 7:**
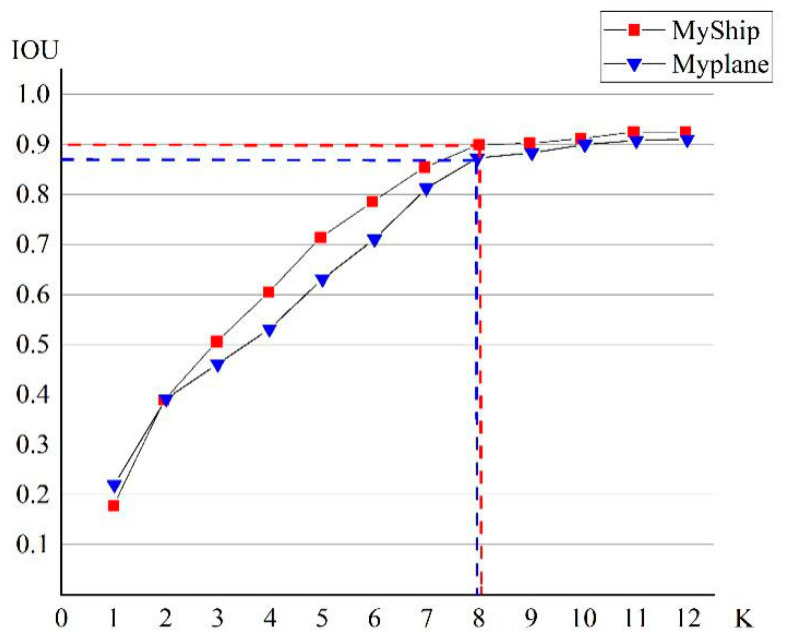
The clustering results of MyPlane and Myship datasets.

An airplane target tends to be square while a ship target is elongated, which makes the shape of the bounding box of the two quite different. The original prior frame size of YOLOv3 is relatively fixed, mainly used for general target detection. Therefore, when using the aerial remote sensing datasets MyPlane and MyShip to detect small targets, the number of anchor boxes needed to be readjusted. The K-means algorithm [25] is a typical distance-based clustering algorithm, which can determine the similarity of samples based on the distance between each sample, gathering similar samples to form a cluster as the final goal, and using distance to measure similarity—the shorter the distance, the higher the similarity. The traditional K-means algorithm generally uses Euclidean distance to measure the distance, which can directly cluster the width and height of the predicted bounding box and obtain K anchor boxes at the same time. However, when the size of the bounding box is large, significant errors occur. Therefore, YOLOv3 [9] introduces the IOU value of the predicted box and the anchor box into the K-means algorithm to determine the anchor box that is most suitable for the existing dataset to improve the detection accuracy of the target. By calculating the IOU values of the two boxes, the degree of similarity between the two boxes can be obtained. In order to better represent the error value, the formula of distance parameter *d* can be defined as follows:

(12) d(box,centroid)=1−IOU(box,centroid),

where box represents the sample and centroid represents the center of the cluster. The smaller the *d*, the more similar the two boxes. We set the range of K from 1 to 12. The IOU values corresponding to the different cluster numbers of the two datasets are shown in Figure 7.

When K = 8, the curves of both datasets tend to be stable. Considering the adjustment of the prediction scale of the network, each anchor box can be equally assigned two prior anchor boxes. The final sizes of the anchor boxes for the MyPlane dataset are (132, 238), (244, 296), (118, 110), (84, 102), (54, 46), (80, 74), (14, 12), (20, 16). Similarly, on the MyShip dataset, the sizes of the anchor boxes obtained by clustering are (126, 306), (86, 174), (58, 120), (44, 92), (28, 54), (24, 42), (26, 10), (16, 12). As the size of the scale is inversely proportional to the range of the receptive field, the receptive field of the 13 × 13 scale is the most sensitive to large targets. 

**Figure 8:**
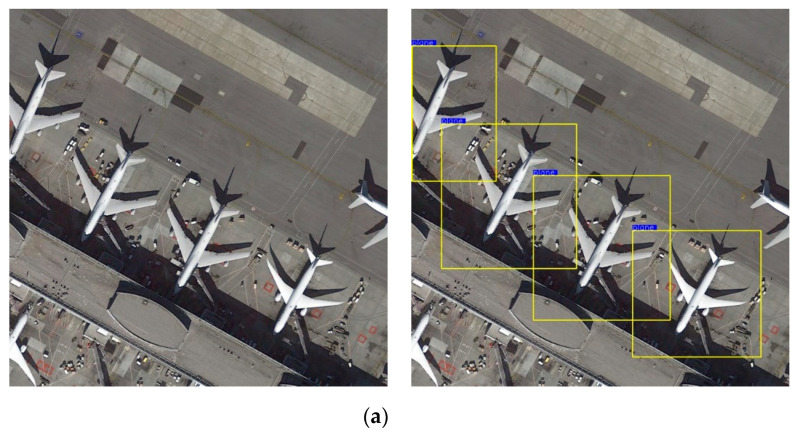

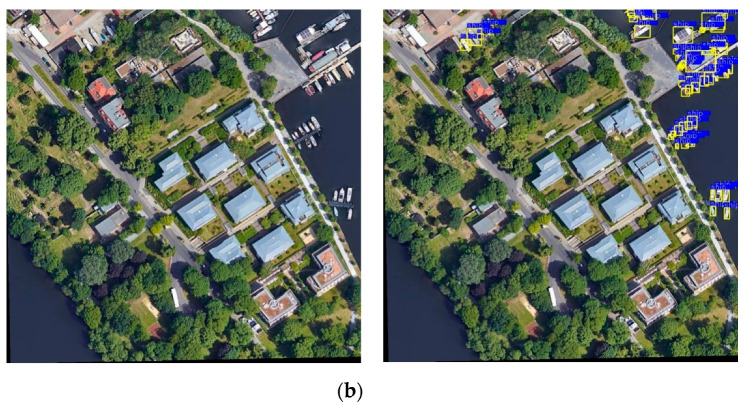
The final calibration and clustering results of the datasets: (**a**) airplanes; (**b**) ships.

The final calibration and clustering results are shown in Figure 8. It can be seen that the size of the airplane targets in the MyPlane dataset images varies, and the general size of the prior box tends to be square; while the size of the MyShip dataset tends to be more elongated. The results are consistent with the actual situation, and it can be seen that a reasonable choice of the corresponding number and the size of anchor boxes helps improve the precise positioning and detection of specific targets.

#### 3.1.3. Model Training

We used four different network models for the training of the two specific target datasets. Since it is only a difference in dataset types, the MyPlane dataset will be used as an example to introduce the experimental process in detail. The four experimental procedures on the MyShip dataset are similar. The training process for the four models is roughly the same, and here we will mainly introduce the training process of the improved model.

**Figure 9:**
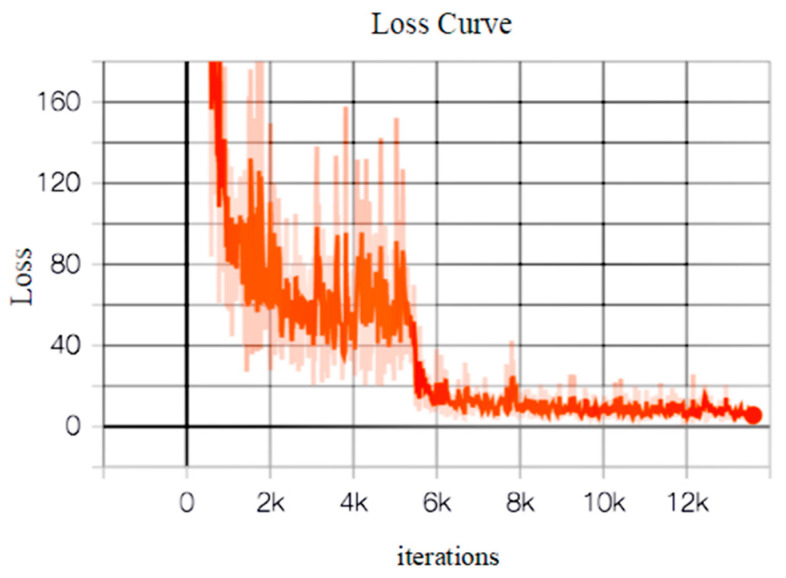
The training loss function graph of improved model on the MyPlane dataset.

The programming language of the algorithm in this paper was Python 3.5.2, the deep learning framework was TensorFlow 1.11.0, and the operating system was Ubuntu 16.04. The hardware platform was Intel(R) Core(TM) i7-7700K CPU at 4.20 GHz and NVIDIA GTX 1060Ti GPU for accelerating model training. In the self-made datasets, there were 1264 training samples, 368 validation samples, and 736 test samples in the MyPlane datasets; while, there were 1754 training samples, 608 validation samples, and 655 test samples in the MyShip dataset. The model training process was used to conduct preliminary training of remote sensing images using the two self-made datasets and the validation set was used for further validation. The preset training parameters were as follows: the momentum was 0.9, the weight decay was 0.0005, the initial learning rate was 0.001, and the maximum number of iterations was 50,200. The learning rate dropped to 0.0001 after 5500 iterations, and to 0.00001 after 20,000 iterations. We set the batch of the improved model to 3; the training epoch was 50, with 20 rounds in the first phase and 30 rounds in the second phase. The graph of the loss function of the improved model on the MyPlane dataset is shown in Figure 9.

Figure 9 shows that the loss function of the improved model drops significantly when the number of iterations reaches about 5500. After the current 20 rounds of training were completed, the entire network was still underfitting and the loss function was relatively large; the subsequent 30 rounds adjusted the learning rate in time to make the network model fit better to continuously reduce the loss function. By drawing the loss curve, the training process of the network can be observed intuitively.

### 3.2. Experiments and Analysis

The YOLOv3 network is presented in this paper to test the target detection effect of the improved model. The experiment uses Faster R-CNN+Resnet101, Faster R-CNN+VGG16, YOLOv3, and the improved model based on YOLOv3 Network to train and verify the two aerial remote sensing datasets MyPlane and MyShip and consider the detection capabilities of the four network models from the detection result map and multiple evaluation indexes. The background of the original airplane images in the remote sensing images is dim and the number of interfering objects is large, which presents a challenge to the detection work. To effectively evaluate the performance of the network model, the accuracy rate P, the recall rate R, the false alarm rate F, the miss alarm rate M, and the average precision (AP) were selected to evaluate the detection capability of the network model. The formulas are as follows:

(13) $$\begin{array}{l} P=\frac{TP}{TP+FP}\\ R=\frac{TP}{TP+FN}\\ F=\frac{FP}{TP+FP}=1-P\\ M=\frac{FN}{TP+FN}=1-R\\ AP=\int_{0}^{1}P(R)\mathrm{dR} \end{array}\}$$

where True Positive (TP) predicts positive samples as positive samples; True Negative (TN) predicts negative samples as negative samples; False Positive (FP) predicts negative samples as positive samples; False Negative (FN) predicts positive samples as negative samples; P indicates the proportion of samples that are correctly detected in all test results; R indicates the proportion of samples that are correctly detected in all samples; AP is the integral value of the Precision rate-Recall rate curve (P-R curve).

There was a total of 736 images in the test set of the MyPlane dataset, including 7436 real targets of the airplane. There was a total of 655 images in the test set of the MyShip dataset, including 68,054 ship targets. For the four different network models, the test results of the two datasets are shown in Table 1 and Table 2. 

The data results in Table 1 and Table 2 prove that the improved algorithm is more sensitive to small targets. We also compared the performance of the four models on two datasets. The results are shown in Table 3 and Table 4. 

It can be seen from Table 3 and Table 4 that the evaluation indicators of the improved model are significantly better than the other three network models. At the same time, the AP comprehensively reflects the detection capability of the model from the two dimensions of accuracy and recall. The larger the AP value, the better the detection. The AP value of the improved model using the MyPlane dataset is 94.29%, compared to the other three network models, which have AP values 12.65%, 13%, and 2.69% lower; the AP value for MyShip dataset is 93.13%, which is 39.75%, 41.83%, and 1.81% higher than the other three network models.

Compared with the improved algorithm, the false detection rate and the missed detection rate of the first three models are higher. This is because the Faster R-CNN+Resnet101 and Faster R-CNN+VGG16 network models do not import multi-scale detection, which leads to lower sensitivity to small targets. By introducing multi-scale detection in the YOLOv3 model, the detection accuracy is higher. However, the scale size and the number of anchor boxes selected in the YOLOv3 model are based on the COCO dataset, which is not very consistent with the data situation of the aerial remote sensing dataset with the majority of small targets. The number of YOLOv3 false detections has increased. We compared the performance of each part of the improved model on two datasets. The results are shown in Table 5 and Table 6. 

The results of Table 5 and Table 6 show that the addition of the 104 × 104 detection scale enables the network to have smaller receptive field feature information. In addition, the overall loss function increases the L2 regularization constraint, which effectively avoids the occurrence of overfitting. Therefore, these two improvements have a certain promotion effect on the detection accuracy of small targets, and the AP and Recall values are improved. At the same time, a reasonable number of prior anchor boxes are set to enhance the ability of network model detection for specific small targets. It can be seen from the data results that the detection effect is slightly improved. 

**Figure 10:**
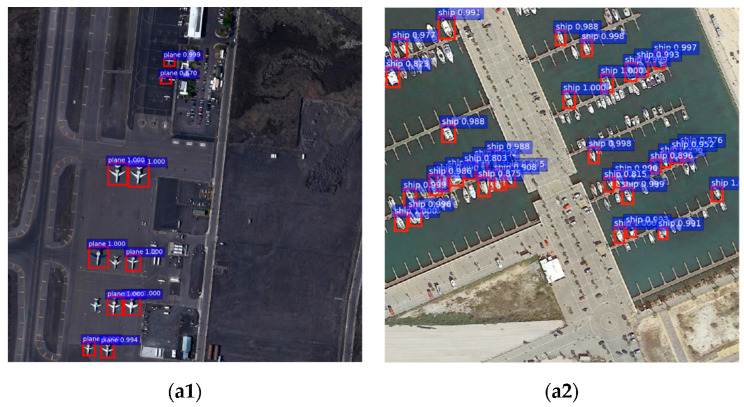

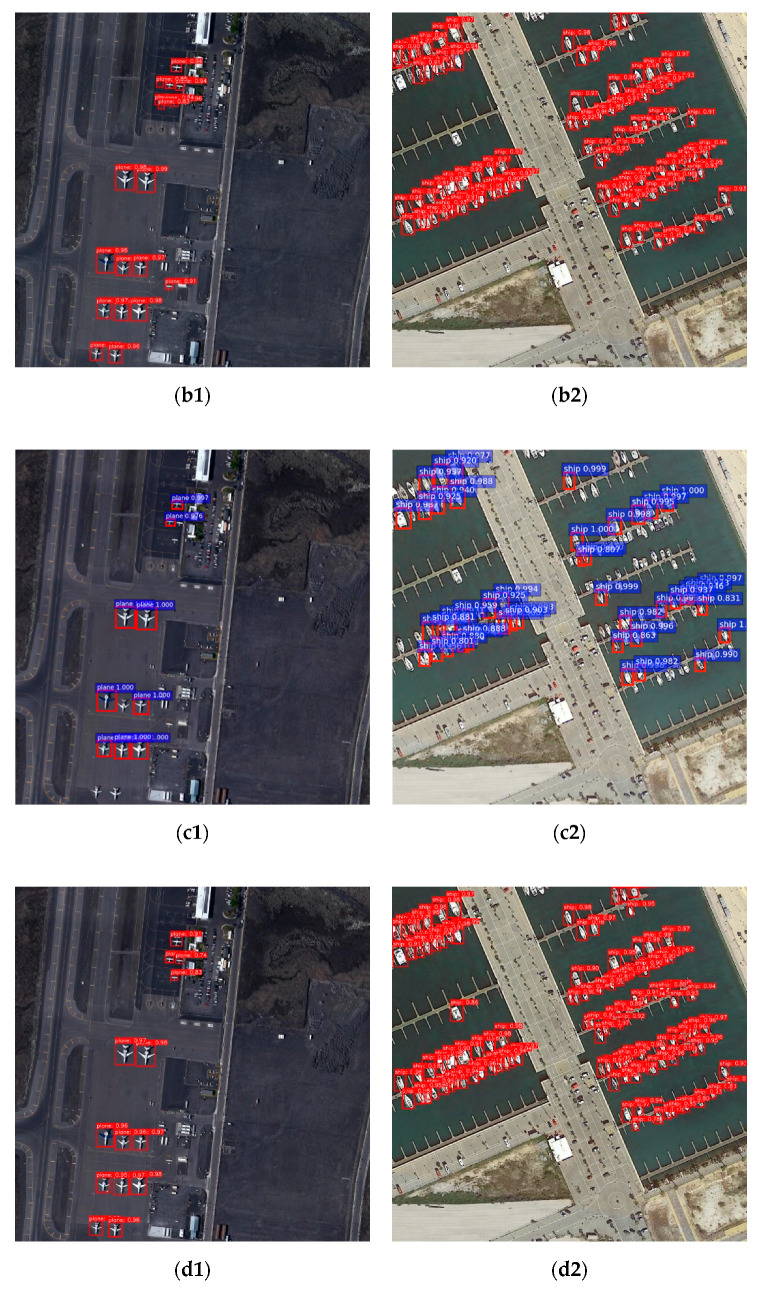
The detection results of different algorithms on two datasets. (**a1**–**d1**): MyPlane dataset; (**a2**–**d2**): MyShip dataset. (**a1**,**a2**) are the detection results of the Faster R-CNN+Resnet101 model; (**b1**,**b2**) are the detection results of the Faster R-CNN+VGG16 model; (**c1**,**c2**) are the detection results of the YOLOv3 model; (**d1**,**d2**) are the detection results of the proposed model in this paper.

Through the above experiments, the results of the algorithm mentioned in this article are shown in Figure 10.

Whether from the index parameters or the actual detection result diagram, the network comprehensive detection capability of the improved model proposed by us is improved. The detection sensitivity of the model to dim small targets is also enhanced. Especially in the MyShip dataset, which had mostly small targets, the advantage and characteristic of the improved model with strong detection sensitivity to small targets was perfectly demonstrated.

## 4. Conclusions

As important strategic resources and means of transportation, airplanes and ships have a practical value that cannot be ignored in the study of aerial remote sensing images. The background of aerial remote sensing images is complex, and the target sizes are diverse. Traditional target detection algorithms cannot accurately extract target features for these objects. We proposed an improved detection algorithm based on the YOLOv3 network, which acquires the specific target samples from the DOTA dataset and resets the number of corresponding anchor boxes to enhance the detection ability. Then, the detection scale was increased to obtain a smaller receptive field and improve the positioning accuracy of small targets. To avoid model overfitting, an L2 regularization term was added to the loss function to reduce the false detection rate. The experimental results show that the proposed model has an improvement of about 1–3% in the accuracy of evaluation indexes in small target detection tasks compared with other algorithms, which effectively improves the ability of the network. By evaluating the optimization of the proposed network model of small targets under two different complex backgrounds (i.e., airplanes and ships), this study has practical significance for the research of aerial remote sensing image detection technology.

## Figures and Tables

**Table 1:** Detection results of four models on the MyPlane dataset.

Network Model	Real Targets	TP	TN	Test Time/s
Faster R-CNN+Resnet101	7436	6399	1133	147.2
Faster R-CNN+VGG16	7436	6383	1145	105.1
YOLOv3	7436	6976	911	25.4
Improved algorithm	7436	7056	708	28.3

**Table 2:** Detection results of four models on the MyShip dataset.

Network Model	Real Targets	TP	TN	Test Time/s
Faster R-CNN+Resnet101	68,054	39,419	10,116	131.0
Faster R-CNN+VGG16	68,054	38,494	10,523	93.6
YOLOv3	68,054	62,761	9413	23.4
Improved algorithm	68,054	63,856	9261	26.2

**Table 3:** Performance comparison of four models on the MyPlane dataset.

Network Model	Precision/%	False/%	Miss/%	AP/%	Train Time/h
Faster R-CNN+Resnet101	84.96	15.04	13.95	81.64	11
Faster R-CNN+VGG16	84.79	15.21	14.16	81.29	9
YOLOv3	88.45	11.55	6.19	91.60	3
Improved algorithm	90.88	9.12	5.11	94.29	5

**Table 4:** Performance comparison of four models on the MyShip dataset.

Network Model	Precision/%	False/%	Miss/%	AP/%	Train Time/h
Faster R-CNN+Resnet101	79.59	20.41	42.08	53.38	14
Faster R-CNN+VGG16	78.53	21.47	43.44	51.30	11
YOLOv3	86.96	13.04	7.78	91.32	5
Improved algorithm	87.33	12.67	6.17	93.13	7

**Table 5:** Performance comparison of each part of the improved model on the MyPlane dataset.

Network Model	104 × 104 Scale	Loss_L2_	Anchor Setting	AP/%	Recall/%
YOLOv3(1)			√	92.17	94.01
YOLOv3(2)	√	√		93.82	94.65
YOLOv3(3)	√	√	√	94.29	94.89

**Table 6:** Performance comparison of each part of the improved model on the MyShip dataset.

Network Model	104 × 104 Scale	Loss_L2_	Anchor Setting	AP/%	Recall/%
YOLOv3(1)			√	91.70	92.54
YOLOv3(2)	√	√		92.75	93.51
YOLOv3(3)	√	√	√	93.13	93.83
